# The relationship between person-vocation fit and burnout among resident physicians: A single-center cross-sectional survey

**DOI:** 10.1371/journal.pone.0324707

**Published:** 2025-06-02

**Authors:** Yaotian Fan, Yue Zhang, Guorong Pu, Qian Zhao, Yuanning Xu, Qian Li, Ke Lin, Zhen You

**Affiliations:** 1 General Practice Medicine Center, West China Hospital, Sichuan University, Chengdu, Sichuan, China; 2 Department of Surgery Teaching and Research Office, West China Hospital, Sichuan University, Chengdu, Sichuan, China; 3 Department of Cardiology, West China Hospital, Sichuan University, Chengdu, Sichuan, China; 4 Anesthesia&Operation Center, West China Hospital, Sichuan University, Chengdu, Sichuan, China; 5 Department of Cardiovascular Surgery, West China Hospital, Sichuan University, Chengdu, Sichuan, China; 6 Division of Biliary Tract Surgery, Department of General Surgery, West China Hospital, Sichuan University, Chengdu, Sichuan, China; West University of Timisoara: Universitatea de Vest din Timisoara, ROMANIA

## Abstract

**Background:**

Burnout among resident physicians affects their physical and mental health as well as the quality of healthcare. Person-Vocation Fit (P-V Fit) may play a key role in preventing burnout. This study aims to explore the relationship between P-V Fit and burnout among resident physicians.

**Method:**

A cross-sectional survey was conducted, including demographic information, work factors, burnout assessment using the Maslach Burnout Inventory-Human Services Survey (MBI-HSS), and the Person-Vocation Fit (P-V Fit) scale. Data were analyzed using logistic and linear regression with SPSS-27.

**Results:**

636 resident physicians (response rate 80%) participated in the survey. The mean age was 25.6 ± 2.3 years, 52.5% were male, 90.6% were unmarried, and 55.7% had a bachelor’s degree. The overall burnout rate was 46.07%, with mean scores for emotional exhaustion, depersonalization, and personal accomplishment of 30.5 ± 11.9, 12.9 ± 7.4, and 28.5 ± 9.6, respectively. For each one-point increase in the P-V Fit score, the overall likelihood of burnout decreased by 19% (OR, 0.81;95%CI, 0.76–0.87;P < 0.001), emotional exhaustion decreased by 0.85 points (95%CI, -1.10 to -0.59), depersonalization decreased by 0.55 points (95%CI, -0.73 to -0.38), and personal accomplishment increased by 1.34 points (95%CI, 1.12–1.56)(all P < 0.001). For each additional hour of work, the risk of burnout increased by 33% (OR, 1.33;95%CI, 1.12–1.59;P = 0.001).

**Conclusion:**

Improving Person-Vocation Fit is an effective strategy for preventing burnout among resident physicians. Residency training should focus on vocational compatibility assessment, develop differentiated support strategies, and arrange reasonable workloads.

## Introduction

Physicians play a crucial role in society as guardians of health, dedicated not only to treating diseases but also to preventing illness and improving public health. However, the medical profession is characterized by high-intensity work pressure, leading to mental health issues among doctors such as burnout, anxiety, depression, and suicidal tendencies [1]. According to the World Health Organization (WHO), by 2030, there will be a global shortage of nearly 14 million healthcare workers, with physicians accounting for approximately 16%, which will significantly impact public health [2]. Resident physicians, as an essential reserve force for future independent practitioners, play a vital role in maintaining the sustainability of the entire healthcare system through their professional development and well-being

In recent years, burnout among healthcare professionals, including resident physicians, has attracted increasing attention [3–6]. Previous studies have indicated that physicians experience peak burnout symptoms during residency training, with burnout rates significantly higher than in other professional groups [7,8]. Residency training represents the critical pathway for medical students to develop into qualified clinicians. During this early career stage, residents must not only master extensive medical knowledge and clinical skills but also face long working hours, research tasks, and clinical decision-making responsibilities—multiple pressures that may lead to burnout [9–11]. Burnout, characterized as a psychological state resulting from prolonged poor coping with work stress, manifests as emotional exhaustion, depersonalization, and reduced personal accomplishment [12]. It affects not only the psychological health and quality of life of resident physicians [13–15], but may also impact the quality and safety of patient care [16,17], and even trigger career regret and intention to leave the profession [18,19]. Despite the widespread attention to burnout among resident physicians, effective prevention and intervention remain challenging aspects of medical education and management.

Research has found that the choice of a medical career or residency specialty is influenced by multiple factors, including personal interests, career prospects, and social expectations, with academic interest and personal abilities being the primary determinants [20]. Career development theory suggests that career choice and development are dynamic processes, with individuals facing new challenges and choices at different career stages [21]. The residency phase can be viewed as a critical turning point in a medical career, during which physicians must adapt to highly specialized clinical work while managing tremendous psychological and physical pressures—factors that often profoundly impact their professional identity and commitment [22].The concept of Person-Environment Fit (P-E Fit) originated in occupational psychology, where a good match between individuals and their work environment positively influences job satisfaction and performance [23,24]. As an important branch of P-E Fit, Person-Vocation Fit (P-V Fit) refers to the compatibility between an individual’s needs, abilities, and values with the demands, ability requirements, and values of the vocational environment [25], specifically exploring the match between individuals and the requirements of their chosen profession [24]. Medical students with poor P-V Fit typically experience reduced job satisfaction and professional commitment [26]. Previous studies have shown that low job satisfaction is associated with poor psychological health outcomes in physicians, including burnout [27], while burnout itself is a predictor of poor job satisfaction [28]. Low job satisfaction is also positively correlated with intention to leave the profession [29]. Although numerous studies have examined factors related to burnout, such as excessive workload and work-family conflict [30–32], as well as personal factors like gender, age, and stress-coping abilities [33], the relationship between P-V Fit and burnout among resident physicians has not been adequately explored.

Based on this background, this study aimed to investigate the relationship between Person-Vocation Fit (P-V Fit) and burnout among resident physicians, hoping to provide a new perspective for understanding the issue of burnout in this population.

## Method

### Study design, participants, and procedures

From February 1 to June 30, 2024, we conducted a cross-sectional, anonymous electronic survey using purposive sampling. The director of our training center contacted teaching directors at various training bases to initiate this survey. Teaching directors who agreed to participate distributed the electronic questionnaire link via the online survey platform “Wenjuanxing” (https://www.wjx.cn/) in WeChat groups to recruit participants. To ensure each participant completed the questionnaire only once, the system was configured with IP address and WeChat account restrictions to prevent duplicate submissions. Electronic questionnaires could only be successfully submitted after all survey items were completed, resulting in no missing values in the received questionnaires. We estimated that participants would need at least 120 seconds to complete the questionnaire. On the first page of the electronic questionnaire, potential participants were informed of the research purpose, and submission of the questionnaire represented consent to participate in the study. This research was granted a waiver of informed consent and approved by the Ethics Review Committee of West China Hospital, Sichuan University (2024–183).

### Sample size

We estimated the required sample size using the equation: $n=Z_{\left(\alpha /2\right)}^{2}\times P\times \left(1-P\right)/e^{2}$, where α=0.05. Based on previous study on the burnout rate among Chinese resident physicians [34], P was set at 50%, and  e  at 0.05. Considering a 20% non-response rate, the minimum required sample size for this survey was 480. This study distributed 820 questionnaires, with 660 participants (response rate 80%) completing the survey. After excluding 24 invalid questionnaires, 636 valid questionnaires were included in the final analysis, exceeding the minimum required sample size.

### Survey instruments

#### Demographic characteristics.

This section inquired about participants’ demographic characteristics, including gender, age, marital status, training level (PGY, Post-Graduate Year, including PGY1, 2, or 3), educational background, clinical specialty, and work environment factors, including average monthly night shift frequency since training began, average daily working hours, and daily sleep duration.

#### Burnout.

Burnout was assessed using the Chinese version of the Maslach Burnout Inventory-Human Services Survey (MBI-HSS), which is considered the gold standard for measuring burnout [12]. The MBI-HSS has been widely used in China and demonstrates good internal consistency [35–38]. The inventory includes three subscales: Emotional Exhaustion (EE; nine items), Depersonalization (DP; five items), and Personal Accomplishment (PA; eight items). Responses are scored on a 7-point Likert scale ranging from “never” (0) to “every day” (6). Scores are categorized as follows: low EE ≤ 18, moderate EE 19–26, high EE ≥ 27; low DP ≤ 5, moderate DP 6–9, high DP ≥ 10; high PA ≥ 40, moderate PA 34–39, low PA ≤ 33. Burnout was defined as high levels of burnout on multiple subscales, specifically in the highest tertile for EE and DP and the lowest tertile for PA. Following the recommendations in the latest MBI manual (fourth edition) [39], which advises against accumulating the scores from the three dimensions to form a single burnout score, we analyzed the mean scores for each individual dimension of the burnout scale.

#### Person-vocation fit.

We used the Chinese version of the Person-Vocation Fit (P-V Fit) scale to assess the degree of individual-vocation matching, which has good internal consistency [26]. It contains 3 questions: “1. There is a good fit between your personal interests and the profession you are engaged in”; “2. My skills and abilities are well suited for the vocation that I am currently in.”; “3. When I think about my interests, I sometimes wonder whether I chose the right occupation”. The third question is reverse-scored. Response options for each item range from “strongly disagree” to “strongly agree” on a 7-point Likert scale, with higher scores representing better person-vocation fit.

### Data analysis

Data analysis was performed using SPSS 27.0 (IBM Corp, Armonk, NY, USA). Categorical variables were presented as frequencies and percentages, and continuous variables as means ± standard deviations. Chi-square tests or Fisher’s exact tests were used to analyze differences in burnout across different variables, while t-tests or analysis of variance were used to compare P-V Fit scores between different groups. Pearson correlation analysis was used to examine relationships among continuous variables. Factors influencing burnout (yes/no) were analyzed using backward LR binary logistic regression, with results presented as odds ratios (OR) and 95% confidence intervals. Burnout was defined as simultaneously meeting high emotional exhaustion (≥27 points), high depersonalization (≥10 points), and low personal accomplishment (≤33 points). Factors influencing P-V Fit scores and the three dimensions of burnout were analyzed using backward stepwise multiple linear regression, with results presented as regression coefficients (B) and 95% confidence intervals. Both regression models included demographic characteristics and work factors as independent variables, with categorical variables converted to dummy variables. All models underwent appropriate diagnostic testing, including the Hosmer-Lemeshow test for logistic regression and multicollinearity and residual analysis for linear regression. All statistical tests were two-sided, with p < 0.05 indicating statistical significance.

## Results

### Scale reliability analysis

In this study, the Cronbach’s α coefficient for the entire MBI-HSS was 0.78, with coefficients of 0.90, 0.83, and 0.85 for the three subscales of emotional exhaustion, depersonalization, and personal accomplishment, respectively. The Cronbach’s α coefficient for the Person-Vocation Fit (P-V Fit) scale was 0.72. According to Tavakol and Dennick’s research [40], Cronbach’s α coefficients between 0.70–0.95 are considered acceptable levels of internal consistency. Lower values indicate poor internal consistency of the measurement tool, while values that are too high (>0.95) may suggest item redundancy in the scale. Therefore, all measurement tools used in this study demonstrated acceptable internal consistency.

### Participant characteristics

Of the 820 resident physicians who received the electronic questionnaire invitation, 660 (response rate 80%) completed the survey. After screening, 24 questionnaires were excluded due to duplicate answers, completion time being too short (<120 seconds), or consistent responses to logically reversed items. Finally, 636 valid questionnaires (valid questionnaire rate 96.3%) were analyzed.

The demographic and work characteristics of participants are shown in Table 1. Participants ranged in age from 21–32 years, with a mean age of 25.57 ± 2.26 years. The majority were unmarried (90.6%) males (52.5%), with educational backgrounds primarily at the bachelor’s (55.7%) and master’s (39.5%) levels. Anesthesiology (23.1%) was the most common specialty among surveyed physicians, followed by family medicine and general surgery. Most resident physicians averaged 4.13 ± 1.68 night shifts per month (range: 1–9), worked an average of 10.79 ± 1.20 hours per day (8–12 hours), and slept an average of 5.74 ± 0.82 hours per day (3.5–8.5 hours).

**Table 1:** Burnout Across Demographic Factors Among 636 residents.

Variable	Mean±SD (Range)/N(%)	Burnout, N(%)		P
		No	Yes	
Sex				<0.001
Male	334(52.5)	201(60.2)	133(39.8)	
Female	302(47.5)	142(47)	160(53)	
Age(y)	25.57 ± 2.26(21-32)			0.002
20-22	10(1.6)	9(90)	1(10)	
23-25	362(56.9)	181(50)	181(50)	
26-28	201(31.6)	107(53.2)	94(46.8)	
29-31	40(6.3)	28(70)	12(30)	
>31	23(3.6)	18(78.3)	5(21.7)	
Marital status				0.004
Single	576(90.6)	300(52)	276(48)	
Married	60(9.4)	43(71.7)	17(28.3)	
Academic degree^a^				<0.001
Undergraduate	354(55.7)	173(48.9)	181(51.1)	
Postgraduate	251(39.5)	142(56.6)	109(43.4)	
Doctor	31(4.9)	28(90.4)	3(9.6)	
Speciality				<0.001
General Surgery	102(16)	57(57)	43(43)	
Orthopaedics	69(10.8)	46(66.7)	23(33.3)	
Cardiothoracic surgery	29(4.6)	14(51.9)	13(48.1)	
Urology	42(6.6)	28(66.7)	14(33.3)	
Neurosurgery	24(3.8)	12(52.2)	11(47.8)	
Pediatric surgery	22(3.5)	15(68.2)	7(31.8)	
Plastic surgery	15(2.4)	10(66.7)	5(33.3)	
Cardiology	6(0.9)	5(83.3)	1(16.7)	
Respiratory Medicine	13(2)	8(61.5)	5(38.5)	
Digestive disease	9(1.4)	8(88.9)	1(11.1)	
Nephrology	8(1.3)	6(75)	2(25)	
Endocrinology	4(0.6)	3(75)	1(25)	
Haematology	7(1.1)	5(71.4)	2(28.6)	
Rheumatology	2(0.3)	3(100)	0(0)	
Epidemiology	3(0.5)	1(50)	1(50)	
Presbyatrics	5(0.8)	3(60)	2(40)	
Family medicine	104(16.4)	56(55.4)	45(44.6)	
Emergency medicine	25(3.9)	19(76)	6(24)	
Anesthesiology	147(23.1)	42(28.6)	105(71.4)	
Training level (postgraduate year, PGY)				0.95
First-year	239(37.6)	127(53.1)	112(46.9)	
Second-year	198(31.1)	108(54.5)	90(45.5)	
Third-year	199(31.3)	108(54.3)	91(45.7)	
Training identity^b^				0.002
Social trainee	196(17.5)	93(47.4)	103(52.6)	
Commissioned by foreign hospital	103(18.9)	56(54.4)	47(45.6)	
Commissioned by our hospital	28(5.6)	24(85.7)	4(14.3)	
Professional master graduate student	309(58)	170(55)	139(45)	
Night shifts per month(n)	4.13 ± 1.68(1-9)			<0.001
1-2	98(7.3)	39(39.8)	59(60.2)	
3-5	440(78.2)	262(59.5)	178(40.5)	
6-8	80(14.1)	39(48.8)	41(51.2)	
>8	18(0.4)	3(16.7)	15(83.3)	
Hours worked per day (h)	10.79 ± 1.20(8-12)			<0.001
< 9	31(6.2)	23(74.2)	8(25.8)	
9-10	173(35.1)	115(66.5)	58(33.5)	
10-11	144(25.8)	83(57.6)	61(42.4)	
>11	288(32.9)	122(42.4)	166(57.6)	
Hours slept per day (h)	5.74 ± 0.82(3.5-8.5)			0.03
< 4	9(1.4)	3(33.3)	6(66.7)	
4-5	100(15.7)	50(50)	50(50)	
5-6	277(43.6)	136(49)	141(51)	
6-7	233(36.6)	144(61.8)	89(38.2)	
>7	17(2.7)	10(58.8)	7(41.2)	
P-V Fit score	14.18 ± 3.12	15.04(3.06)	13.17(2.87)	<0.001

Abbreviation: P-V Fit, Person-Vocation Fit(score ranges from 3 to 21). N, Number.%, Percentage. P, P Value.SD, standard deviation.

^a^1. Undergraduate: Equivalent to a Bachelor’s degree internationally. Medical undergraduate education in China typically requires 5 years, longer than the 4-year programs for most other majors. Graduates receive a Bachelor of Medicine degree, possess basic medical practice skills, and this serves as the starting point for becoming a registered physician. 2. Postgraduate: Equivalent to a Master’s degree internationally. Medical Master’s programs in China usually take 3 years to complete, during which students undergo both clinical training and research work. Resident physicians with Master’s degrees typically possess more specialized skills. 3. Doctor: Equivalent to a Doctoral degree internationally. As the highest academic qualification in Chinese medical education, it typically takes 3–5 years to complete. Doctoral students not only receive advanced specialty training but must also complete original research work. Resident physicians with doctoral degrees often play more central roles in hospitals and may have more career development opportunities.

^b^In China, there are various types of residency training participants. Some physicians receive training after completing five years of undergraduate medical education, and these persons are classified as “social trainee”. Some physicians are at the master’s level in conjunction with the program, these people are classified as “professional master student”, other physicians have been trained after they have engaged in clinical work, these are called “commissioned trainee”, including personnel affiliated with the training institution of the hospital and personnel of the outside hospital. The educational background of these trainees can range from a bachelor’s degree to a master’s degree or even a doctorate.

### Burnout and person-vocation fit distribution

The overall burnout rate among resident physicians surveyed in this study was 46.07%. The mean (SD) scores for emotional exhaustion, depersonalization, and personal accomplishment were 30.48(11.89), 12.91(7.40), and 28.46(9.58), respectively. The mean (SD) score for Person-Vocation Fit was 14.18(3.12). Person-Vocation Fit was associated with burnout (Table 1), with resident physicians without burnout having a mean (SD) P-V Fit score of 15.04(3.06), while those with burnout had a mean (SD) P-V Fit score of 13.17(2.87) (p < 0.001). Person-Vocation Fit scores did not differ greatly across demographic characteristics, though male residents and those with doctoral degrees had slightly higher P-V Fit scores (Table 2) and lower overall burnout rates (Table 1). Among specialties, plastic surgery residents had the highest P-V Fit scores, while family medicine residents had the lowest P-V Fit scores (Table 2) and higher overall burnout rates (Table 1).

**Table 2:** P-V Fit Scores Across Demographic Factors Among 636 residents.

Variable	P-V Fit score, mean(SD)	t/F-Value^a^	P
Sex		t = 3.20	0.001
Male	14.55(3.20)		
Female	13.76(2.97)		
Age, y		F = 0.87	0.47
20-22	15.10(2.02)		
23-25	14.22(3.11)		
26-28	13.93(3.16)		
29-31	14.47(3.47)		
>33	14.82(2.51)		
Marital status		t = -1.61	0.11
Single	14.11(3.12)		
Married	14.80(3.03)		
Academic degree		F = 3.55	0.014
Undergraduate	13.87(3.23)		
Postgraduate	14.46(2.98)		
Doctor	15.35(2.36)		
Speciality		F = 3.54	<0.001
General Surgery	14.83(2.69)		
Orthopaedics	15.28(2.59)		
Cardiothoracic surgery	14.13(3.72)		
Urology	15.04(2.72)		
Neurosurgery	14.62(2.33)		
Pediatric surgery	14.54(3.21)		
Plastic surgery	16.46(3.02)		
Cardiology	14.66(3.20)		
Respiratory Medicine	13.76(2.80)		
Digestive disease	15.11(1.16)		
Nephrology	13.62(1.84)		
Endocrinology	15.00(1.41)		
Haematology	14.57(2.82)		
Rheumatology	16.00(1.20)		
Epidemiology	15.33(2.31)		
Presbyatrics	14.20(2.77)		
Family medicine	12.89(3.05)		
Emergency medicine	14.84(3.15)		
Anesthesiology	13.29(3.49)		
Training level (postgraduate year, PGY)		F = 1.01	0.36
First-year	14.41(2.75)		
Second-year	14.04(3.17)		
Third-year	14.05(3.47)		
Training identity		F = 8.03	<0.001
Social trainee^b^	13.68(3.46)		
Commissioned by foreign hospital	13.34(3.01)		
Commissioned by our hospital	15.21(2.55)		
Professional master graduate student	14.68(2.86)		
Night shifts per month, No.		F = 5.17	0.002
1-2	13.35(3.54)		
3-5	14.43(2.87)		
6-8	14.21(3.05)		
>8	12.44(5.22)		
Hours worked per day, h		F = 0.43	0.72
< 9	14.12(3.12)		
9-10	14.30(2.90)		
10-11	14.34(2.88)		
>11	14.03(3.35)		
Hours slept per day, h		F = 1.85	0.11
< 4	15.22(5.51)		
4-5	14.58(3.42)		
5-6	14.35(2.86)		
6-7	13.82(3.08)		
>7	13.47(3.84)		

Abbreviation: P-V Fit, Person-Vocation Fit(score ranges from 3 to 21). SD, standard deviation.P, P Value Mean (SD) P-V Fit score, 14.18(3.12).

^a^Independent t-tests and one-way analysis of variance for binary and categorical predictor variables.

^b^In China, Some physicians receive training after completing five years of undergraduate medical education, and these persons are classified as “social trainee”.

### Multivariate regression analysis

Multivariate linear regression analysis (Table 3) showed that resident physicians with burnout had P-V Fit scores 1.71 points lower than those without burnout (95%CI, -0.71 to -0.16; P < 0.001). Interestingly, for each additional hour of sleep, the P-V Fit score decreased by 0.44 points (95%CI, -0.72 to -0.17; P = 0.002). Logistic regression analysis (Table 4) indicated that working hours were an important predictor of burnout, with each additional hour increasing the overall burnout rate by 33% (OR, 1.33; 95%CI, 1.12–1.59; P = 0.001). Simultaneously, for each one-point increase in P-V Fit score, the overall burnout rate decreased by 19% (OR, 0.81; 95%CI, 0.76–0.87; P < 0.001).

**Table 3:** Multivariable Linear Regression Model of the Residents P-V Fit score^a^.

Variables	P-V Fit score	
	β(95%)CI	P
Speciality		
General Surgery	1(reference)	
Plastic surgery	1.75(0.26-3.25)	0.02
Family medicine	-1.62(-0.26 to -0.99)	<0.001
Anesthesiology	-0.87(-1.45 to -0.29)	0.003
Hours slept per day(h)	-0.44(-0.72 to -0.17)	0.002
Burnout:(Yes VS.No)	-1.71(-0.71 to -0.16)	<0.001

Abbreviation: P-V Fit, Person-Vocation Fit(score ranges from 3 to 21). β, Beta Coefficient. CI, Confidence Interval. P, P Value.

Mean(SD) P-V Fit score, 14.18(3.12).

^a^The R-squared of this model is 0.13.

**Table 4:** Multivariable Logistic Regression Model of the Burnout^a^.

Variables	Burnout(Yes VS.No)	
	OR(95%)CI	P
Speciality		
General Surgery	1(reference)	
Orthopaedics	0.55(0.28-1.09)	0.09
Cardiothoracic surgery	1.05.(0.43-2.57)	0.89
Urology	0.59(0.26-1.31)	0.19
Neurosurgery	0.89(0.34-2.31)	0.82
Pediatric surgery	0.64(0.21-1.89)	0.41
Plastic surgery	0.93(0.27-3.17)	0.91
Cardiology	0.39(0.03-4.18)	0.43
Respiratory Medicine	1.19(0.32-4.37)	0.78
Digestive disease	0.22(0.02-1.97)	0.17
Nephrology	0.33(0.06-1.85)	0.21
Endocrinology	0.45(0.05-4.63)	0.51
Haematology	1.24(0.19-8.15)	0.81
Rheumatology	0.32(0.06-2.31)	0.56
Epidemiology	1.33(0.10-17.82)	0.83
Presbyatrics	0.78(0.11-5.49)	0.81
Family medicine	1.26(0.57-2.76)	0.56
Emergency medicine	0.47(0.16-1.43)	0.18
Anesthesiology	2.84(1.38-5.80)	0.004
Training identity		
Social trainee	1(reference)	
Commissioned by foreign hospital	1.22(0.63-2.35)	0.53
Commissioned by our hospital	0.47(0.13-1.71)	0.25
Professional master graduate student	2.05(1.16-3.60)	0.01
Hours worked per day (h)	1.33(1.12-1.59)	0.001
P-V Fit score	0.81(0.76-0.87)	<0.001

Abbreviation: P-V Fit, Person-Vocation Fit(score ranges from 3 to 21). OR, Odds Ratio. CI, Confidence Interval. P, P Value.

Mean(SD) P-V Fit score, 14.18(3.12).

^a^The Hosmer-Lemeshow goodness-of-fit test resulted in a p-value of 0.54, indicating the model fits well.

Multivariate linear regression analysis also showed (S1 Table) that for each one-point increase in P-V Fit score, emotional exhaustion scores decreased by 0.85 points (95%CI, -1.10 to -0.59; P < 0.001), depersonalization decreased by 0.55 points (95%CI, -0.73 to -0.38; P < 0.001), and personal accomplishment scores increased by 1.34 points (95%CI, 1.12–1.56; P < 0.001). Working and sleeping hours also significantly affected the three dimensions of burnout. For each additional hour of work, emotional exhaustion increased by 1.48 points (95%CI, 0.71–2.26; P < 0.001), depersonalization increased by 0.63 points (95%CI, 0.11–1.14; P = 0.02), and personal accomplishment decreased by 0.62 points (95%CI, -1.26–0.02; P = 0.05). For each additional hour of sleep, emotional exhaustion decreased by 2.82 points (95%CI, -3.83to-1.81; P < 0.001), depersonalization decreased by 1.35 points (95%CI, -2.02to-0.69;P < 0.001), and personal accomplishment increased by 0.73 points (95%CI, -0.10–1.57; P = 0.08).

### Correlation analysis

Correlation analysis (Table 5) showed that Person-Vocation Fit was negatively correlated with emotional exhaustion (r = -0.273, p < 0.001) and depersonalization (r = -0.263, p < 0.001), and positively correlated with personal accomplishment (r = 0.458, p < 0.001). Daily working hours were positively correlated with emotional exhaustion (r = 0.337, p < 0.001) and depersonalization (r = 0.245, p < 0.001), while sleep duration was negatively correlated with these dimensions (r = -0.238 and r = -0.177, respectively, p < 0.001).

**Table 5:** Correlation matrix of burnout dimensions, P-V Fit and related Variables.

Variable	Ee	Dp	Pa	P-V Fit score
Ee	1	0.788***	-0.411***	-0.273***
Dp	0.788***	1	-0.406***	-0.263***
Pa	-0.411***	-0.406***	1	0.458***
P-V Fit score	-0.273***	-0.263***	0.458***	1
Age(y)	-0.13**	-0.139***	0.009	0.003
Night shifts per month(n)	0.039	0.032	0.03	0.012
Hours worked per day (h)	0.337***	0.245***	-0.159***	-0.034
Hours slept per day (h)	-0.238***	-0.177***	0.049	-0.111**

Abbreviation: Ee, Emotional Exhaustion. Dp, Depersonnalization. Pa, Personal Accomplishment. P-V Fit, Person-Vocation Fit. P, P Value.

**P < 0.01, *** < 0.001.

## Discussion

This study explored the relationship between Person-Vocation Fit (P-V Fit) and burnout among resident physicians. The findings indicate that P-V Fit is closely related to burnout, offering new insights into understanding burnout among resident physicians and suggesting that P-V Fit may be a key factor in preventing burnout during the early stages of a medical career.

Firstly, the burnout rate among resident physicians in this study was 46.07%, indicating that nearly half of residents experienced burnout during their training. This aligns with the global trend of high burnout rates among resident physicians [30], reaffirming the severity of the burnout problem in this population. Female resident physicians, who often face gender discrimination in the workplace, unequal career advancement opportunities, and the need to balance work and family responsibilities, frequently experience higher risk of burnout [41,42]. Single or younger resident physicians, with relatively weaker social support systems, also showed higher burnout rates, consistent with previous findings [7]. Long working hours were identified as a significant factor in resident physician burnout [43]; we found that each additional hour of daily work increased burnout risk by 33% and significantly affected all three dimensions of burnout, suggesting that extended work hours not only increase physical fatigue but may also lead to psychological stress accumulation and emotional exhaustion. Sleep deprivation was likewise an important predictor of burnout, with daily sleep duration showing significant negative correlations with emotional exhaustion (β = -2.82) and depersonalization (β = -1.35), highlighting the protective effect of adequate sleep on resident physicians’ mental health [44].

The most prominent finding of this study is the critical protective role of P-V Fit in preventing burnout. Multivariate analysis showed that for each one-point increase in P-V Fit, resident physicians’ risk of burnout decreased by 19%, with significant effects on all three dimensions of burnout: emotional exhaustion scores decreased by 0.85 points, depersonalization scores decreased by 0.55 points, and personal accomplishment scores increased by 1.34 points. Emotional exhaustion, as the core component of burnout, is often the first symptom residents experience when facing high-intensity work and stress. The improvement in P-V Fit effectively alleviates emotional exhaustion among resident physicians, suggesting that when residents feel highly matched with their profession, they can better manage and cope with work stress and maintain emotional stability. Depersonalization reflects the residents’ apathy and sense of alienation towards patients. Higher P-V Fit indicates a stronger sense of identification with and commitment to their professional role, reducing the likelihood of a sense of detachment at work.Personal accomplishment is the third dimension of burnout, with P-V Fit having the most significant impact (β = 1.34), indicating that when resident physicians perceive a high congruence between their abilities and professional requirements, they more easily derive satisfaction and a sense of achievement from their work, thereby reducing the occurrence of burnout. High accomplishment not only enhances resident physicians’ professional identity but also improves their positive expectations for future career development. Overall, these findings suggest that P-V Fit influences resident physician burnout through multiple pathways. It mitigates the negative dimensions, such as emotional exhaustion and depersonalization, while enhancing the positive dimension of personal accomplishment, thus reducing the overall risk of burnout. This highlights the importance of improving P-V Fit as a strategy to prevent burnout.

Secondly, our analysis of Person-Vocation Fit revealed that family medicine and anesthesiology residents had lower P-V Fit scores, while plastic surgery residents had higher scores. These differences may reflect the impact of different specialties on resident physicians’ person-vocation fit. The lower P-V Fit scores among family medicine residents may be related to the broad and complex nature of their work. Family medicine involves the diagnosis and management of a wide range of diseases and patient needs, which can make it difficult for residents to achieve a strong sense of professional accomplishment. Additionally, the heavy workload and the need for long-term patient management in family medicine may further decrease residents’ sense of fit with their profession and increase their risk of burnout [45]. Anesthesiologists not only scored lower on P-V Fit but also performed poorly on all three dimensions of burnout, with a burnout risk 2.84 times that of general surgery residents. This may be related to the unique nature of anesthesiology work, including the need for high-intensity vigilance, limited patient contact time, and difficulty in gaining recognition for work achievements [46,47]. In contrast, plastic surgery residents had higher P-V Fit scores, possibly due to the highly technical and rewarding nature of their specialty. Plastic surgery often involves complex surgical techniques and specialized clinical decision-making, which may enhance residents’ sense of professional identity and accomplishment [48], contributing to higher P-V Fit scores and lower rates of burnout.Additionally, the relationship between workload and P-V Fit presented a complex pattern. The study showed that residents with 3–5 night shifts per month had the highest P-V Fit scores (14.43 ± 2.87), which significantly decreased when exceeding 8 shifts (12.44 ± 5.22). This suggests that moderate shift frequency may positively impact resident physicians’ person-vocation fit, but excessive shifts may lead to increased fatigue and burnout. Excessive night shifts exacerbate the physical and psychological stress of residents, making it difficult for them to maintain a high sense of P-V Fit. Prolonged intense work not only affects residents’ physical health but may also lead to perceived overload, reducing job satisfaction and personal accomplishment [49]. The role of sleep duration in P-V Fit is also noteworthy. Although this study found some contradiction between sleep duration and P-V Fit—multivariate analysis showed a negative correlation (β = -0.44), suggesting that residents who sacrifice sleep time might have higher person-vocation fit—this may reflect that highly professionally engaged physicians are willing to sacrifice sleep time while maintaining a strong sense of professional identity. However, this pattern warrants caution, as physicians with insufficient sleep (<4 hours) had a burnout rate as high as 66.7%, indicating that short-term high engagement may lead to increased long-term burnout risk. This study also found that burnout significantly affected resident physicians’ P-V Fit scores, with burnout residents scoring 1.71 points lower than non-burnout residents. This suggests that when resident physicians experience burnout, they are more likely to feel mismatched with their profession, which in turn may exacerbate their burnout, forming a potential vicious cycle. Therefore, reducing burnout not only helps improve resident physicians’ job satisfaction but also enhances their person-vocation fit, creating a positive work state.

The findings of this study have important clinical and managerial implications. Firstly, resident physician training program managers should help physicians better understand and assess their professional interests and abilities before entering the residency stage to improve person-vocation fit. Introducing flexible work arrangements and stress management strategies in resident training can help residents better adapt to their professional environment, thereby improving their mental health and job satisfaction. Secondly, the unique characteristics of different specialties should be considered, and personalized support strategies should be developed to enhance residents’ job satisfaction and performance.Finally, healthcare institutions should emphasize work time and sleep management, reasonably arrange shift frequency, and avoid excessive workloads. Although some physicians may demonstrate professional commitment by sacrificing sleep time, the long-term burnout risks associated with such unhealthy work patterns should be cautioned against.

Despite providing new insights into understanding resident physician burnout, this study has some limitations. Firstly, it only examined the relationship between P-V Fit and burnout at a single time point, without measuring the dynamic changes in these factors over time. Therefore, we cannot determine how these factors change over time. Secondly, as a cross-sectional study, it cannot establish a causal relationship between P-V Fit and burnout among resident physicians.Finally, although the study covered resident physicians from multiple departments, the sample primarily came from specific hospitals and may not comprehensively represent all resident physicians in China. Additionally, the purposive sampling method used may have introduced self-selection bias—resident physicians with excessive work pressure or severe burnout may have lacked the time or motivation to participate in the survey. Future research should consider using a multi-center, stratified random sampling design, including resident physician samples from different regions and hospital levels to obtain more representative results.

## Conclusion

This study demonstrates a significant correlation between Person-Vocation Fit and burnout among resident physicians. The findings reveal that for each one-point increase in P-V Fit score, resident physicians’ risk of burnout decreases by 19%, with significant effects on all three dimensions: emotional exhaustion, depersonalization, and personal accomplishment. This indicates that higher Person-Vocation Fit serves as an important protective factor against burnout. Future residency training and management should focus more on enhancing P-V Fit and developing personalized intervention strategies to reduce burnout and promote the professional development and mental health of resident physicians.

## Supporting information

S1 Table Multivariable Linear Regression Model of Factors Influencing Three Dimensions of Burnout Among Residents. (XLSX)

S1 File Original data. (XLSX)
